# *Dyella japonica* Bacteremia in Hemodialysis Patient

**DOI:** 10.3201/eid1308.061204

**Published:** 2007-08

**Authors:** Pattarachai Kiratisin, Premwadee Kowwigkai, Supanit Pattanachaiwit, Anucha Apisarnthanarak, Amornrut Leelaporn

**Affiliations:** * Mahidol University, Bangkok, Thailand; † Thammasat University Hospital, Prathumthani, Thailand

**Keywords:** Dyella japonica, Xanthomonadaceae, soil bacteria, hemodialysis, bacteremia, letter

**To the Editor:** Patients who receive long-term hemodialysis are at great risk for infection (*1*,*2*), especially bacteremia, which may lead to devastating outcomes (*3*). Environmental bacteria are commonly recovered from dialysis fluid, but their contribution to infection is less evident (*4*). We report a bacteremic episode caused by an unusual soil bacterium, *Dyella japonica.* The patient was a 69-year-old Thai woman who had had end-stage renal disease for 8 months and was receiving hemodialysis twice a week via subclavian double-lumen permanent catheter. Approximately 6 h after hemodialysis, she became febrile. Physical examination showed temperature 38^o^C, respiratory rate 22/min, heart rate 80/min, and blood pressure 130/60 mmHg. The rest of her examination was unremarkable and included normal state of consciousness, clear eyeground (fundus), and absence of a heart murmur. Her catheter was intact without evidence of exit site or catheter infection.

Two blood samples, 1 each from the central line and peripheral line, were injected into BACTEC Aerobic/F bottles and incubated in the BACTEC 9240 system (Becton-Dickinson Diagnostic Systems, Sparks, MD, USA). A catheter-related bacteremia was suspected, and vancomycin (1 g in intravenous drip) was prescribed. Other laboratory findings included a total leukocyte count 14.5 × 10^9^/L (84% neutrophils, 16% lymphocytes), blood urea nitrogen 38 mg/dL, and creatinine 7.9 mg/dL. Urinalysis results were within normal limits. Urine and stool cultures were negative for pathogenic bacteria. The catheter was not removed for culture. On day 4 of incubation, both blood cultures showed growth, which was then placed onto 5% (vol/vol) sheep blood agar for subculture and produced deep yellow colonies. This uniform, gram-negative, oxidase-positive bacterium was not identifiable with manual phenotypic tests and the API 20NE strip (bioMérieux, Durham, NC, USA). It was identified by the Vitek 2 system (bioMérieux) and reported to be *Myroides* sp. with an excellent confidence level (98.7% probability).

To further confirm the identification, we used 16S rDNA analysis. The primer pair forward 5′-AGAGTTTGATCMTGGCTCAG-3′ and reverse 5′-ACGGYTACCTTGTTACGACTT-3′ was used to amplify the 16S rDNA by PCR. DNA extraction and PCR amplification were carried out as described (*5*). The sequence of 16S rDNA amplicon (1,450 bp) was determined after electrophoresis and performed with the 3100 Genetic Analyzer (Applied Biosystems, Foster City, CA, USA) according to the manufacturer’s recommendations. The 16S rDNA sequence of this isolate (strain RB28), deposited in GenBank under accession no. DQ984127, was compared with sequences in GenBank by using the BLAST algorithm (version 2.0; National Center for Biotechnology Information, Bethesda, MD, USA, www.ncbi.nlm.nih.gov/blast). Sequence alignment and distance analysis were performed with Lasergene software (DNASTAR, Inc., Madison, WI, USA). According to the 16S rDNA sequence analysis, our isolate belonged to the family *Xanthomonadaceae* of the Gamma Proteobacteria class; the highest sequence similarity (99.2%) was obtained for *D. japonica* type strain XD53 (*6*). In contrast, RB28 shared <97% sequence similarity to other species of *Dyella* and other genera in this family (data not shown). Organisms within the same species should share >97% of 16S rDNA sequence similarity (*7*). Therefore, this isolate was identified as *D. japonica.* The biochemical profile of RB28 was also most consistent with *D. japonica* (Table)*.*

**Table:** Biochemical characteristics of patient’s isolate RB28 and type strains of *Dyella* species*

Characteristics	Patient’s isolate RB28	*D. japonica* XD53^T^	*D. koreensis* BB4^T^
Oxidase	+	+	+
Catalase	+	+	w
Motility	+	+	+
Acid from			
L-arabinose	–	–	–
D-galactose	–	–	–
D-glucose	+	+	+
D-mannose	+	+	–
D-ribose	–	–	–
D-sucrose	–	–	+
D-xylose	–	–	–
Caprate	–	–	–
Citrate	–	–	–
α-galactosidase	–	–	+
β-*N*-acetyl-glucosaminidase	–	w	+
α-glucosidase	–	w	+

*Data from references (*6*) and (*8*); T, type strain.

MIC values as determined by Etest were amikacin 0.75, cefotaxime 0.064, ceftazidime 0.38, ciprofloxacin <0.002, co-trimoxazole 0.125, gentamicin 1.5, imipenem and meropenem 0.25 mg/L. Because of MIC results, treatment was changed to ceftazidime (1 g intravenously every 8 h). Fever abated within a few days without catheter removal. The patient had a complete recovery with no complications. Follow-up blood cultures 2 and 4 weeks after 14 days of treatment were negative.

The *Dyella* genus comprises 3 species: *D. japonica* (*6*), *D. koreensis* (*8*), and *D. yeojuensis* (*9*). All are soil isolates and have been neither isolated from clinical samples nor reported to cause human infection. Their pathogenicity in humans is unknown. Because of its rapid onset after hemodialysis, the bacteremia in this patient is thought to have been associated with the dialysis procedures. Contaminated dialyzing fluid may have been a source for the organism, and the permanent catheter was likely to have provided an entry. In addition, blood culture bottles could have been contaminated by environmental samples. However, the diagnosis of catheter-related infection could not be definitive because neither catheter tip nor fluid was available for culture. The severity of *D. japonica* bacteremia was difficult to determine because the clinical manifestation was mild and the patient responded well to antimicrobial drug therapy, albeit without catheter removal. This case emphasizes that environmental bacteria can be an emerging threat for hemodialysis patients, who are at risk of acquiring opportunistic infection. In addition, this report demonstrates the usefulness of molecular methods for identifying uncommon isolates.
